# Home Rule for Nurses

**Published:** 1887-12-31

**Authors:** 


### The Hospital

A Weekly Institutional Journal of

**Science, Medicine, and Philanthropy.**

*For Hospitals, Asylums, Medical Practitioners, Students, Nurses, Families, Parishes, Congregations, and the Charitable Public.*

Vol. III. No. 66.] [December 31, 1887.

### Home Rule for Nurses.

The nursing profession has attained a position in this country which places it on solid ground. Few, if any, civilised people exist in the present day who do not value the services and recognise the absolute necessity of the skilled nurse in time of sickness. Thanks to Miss Nightingale, the pioneer of good nursing, and to Mrs. Wardroper, to whose practical good sense and devotion the present position of nursing is mainly due—the care, capacity, and status of nurses, not only in this country, but throughout the civilised world, stand so high at the present time as to leave little to be desired. These results have been gradually achieved by the intelligent encouragement of responsible boards of management, the self-denying exertions of a large number of medical men, and the devotion of good women who have come forward year by year in increasing numbers to devote their talents to alleviating the sufferings of our common humanity. It must further be remembered that the development of nursing, like every other question, has been largely a financial one, and the success already attained would never have been secured without the liberal contributions of generous donors throughout the country. In other words, the present position of nurses and the recognised importance of nursing to the whole community are the result of the united efforts and support of nurses, medical men, hospital managers, and generous philanthropists. If any one of these interests had failed to be represented, nursing must have suffered, and the rapid development of nursing of late years would have been impossible.

We desire to call attention to the above considerations at the close of the present year of Jubilee, because the whole welfare of the nursing profession is threatened with a calamity which may prove dangerous, if not fatal, to the best interests of every member of their body. The present is an age of unrest, in which individuals strive for notoriety to an extent probably beyond anything that the world has witnessed before. As the last hours of the old year pass away evidence accumulates that the new year will see a determined effort made to induce nurses of all grades to band themselves together, or as the phrase is—to free themselves from the trammels which association with generous laymen and hospital managers has imposed upon them—that is to assert their right to a position of absolute independence in all respects. Certain ardent spirits, for reasons best known to themselves, are possessed with a burning desire to establish "Home Rule for Nurses" in their relations to institutions and the public. It is therefore our duty to warn every sensible woman who is connected with the nursing profession to have a care before she allows herself to be persuaded to follow any such dangerous counsels or proposals, which must break up nurses into two distinct classes: First, those who desire to do their work efficiently in the interests of the suffering poor by working in harmony with the constituted authorities who have stood by them to the present time; and secondly, those who prefer to take the opposite course. An *imperium in imperio* has crippled the usefulness and destroyed the efficient position of more than one hospital within the knowledge and recollection of the writer, and should this Home Rule movement be forced to the front, the managers of hospitals and nursing institutions throughout the country may be compelled in self-defence to enact that no member of their staff shall become a member of the new Association. The tendency of everything in the hospital economy of to-day wisely tends towards co-operation and unity of purpose. The ambitious proposals to which we here call attention aim at altering all this, with a view to enabling nurses, by asserting their independence, to assume an attitude towards the constituted authorities which must prove damaging to efficiency of administration, and harmful to the sick who seek relief within the walls of the hospitals and kindred institutions.

A striking example of the feeling to which we have alluded was exhibited at the meeting of The Hospitals Association after the reading of Dr. Steele's paper on the registration of nurses. In an assembly of nearly two hundred persons, rather less than forty were present as an organized body with a definite purpose. They had their recognised leaders and acted together with commendable discipline, although some of them, if judged by their dress, may be declared not to have been members of the nursing profession. Their purpose was to object to The Hospitals Association, which represents every branch of hospital economy having anything to do with the registration of nurses. Although they failed to effect their object, we were at a loss to understand the grounds of this opposition until we had the advantage of learning the facts we have set forth at the commencement of this article. It is not our intention to express any opinion of those who have taken the lead in this new departure; but it is necessary to point out to them, and to all who have the best interests of the nursing profession at heart, that it will be a fatal step to attempt to cut off themselves and nurses as a body from those who are responsible for the management and maintenance of the hospital system in this country.

We would refer our readers to the wise counsel contained in Mrs. Bluett's letter which we published in No. 62, Vol. III., page 168. As she says with great force and truth, "I am persuaded The Hospitals Association (the Council of which consists of representatives of all classes of institutions and all sections of hospital work) is the proper medium of communication with the nursing world, and to act wisely, and to ensure the confidence of the public, they will place the registration scheme under a joint committee of gentlemen and ladies, who are themselves fully trained and certificated nurses." This was the view taken by the large majority of the nurses present at the Guy's Hospital meeting, which defeated the organised attempt to oppose the reference of the registration of nurses to such a committee as Mrs. Bluett proposed. The Hospitals Association was authorised to communicate with the Medical Council, and failing them, to elaborate a scheme for submission to a representative meeting of the nursing profession. The committee has yet to be completed, but it ought to include representatives of every interest, which we have shown to have exercised a material influence in raising the profession of nursing to the position it occupies to-day. No doubt such an invitation, if properly made, will be cordially accepted, and so we may hope to secure in the end a scheme for the registration of nurses which will commend itself to the judgment and acceptance of everybody interested in this important question.

We do not wish to be misunderstood as to the plan upon which we think the registation of nurses ought to be conducted. The function of the Council of The Hospitals Association as a body representative of all the interests involved is clear. It is not necessarily any part of their work to undertake the duties and responsibility of registering nurses, but it is their function, if they are to further any useful purpose, to take such action on questions of this character as will secure the expression of the views of all who have a right to be heard on the subject, and to afford such assistance as will best promote the solution of such a problem as this.

There can be no doubt that it would be well if all the nurse-training institutions would combine, with the view of securing an identical system and the establishment of a register on a basis which would secure their confidence and support. Whether (1) a separate and distinct board or other organisation should be constituted for the purpose of examining candidates for nursing certificates, and keeping a register of trained nurses; or whether (2) each nursing school should examine its own pupils and grant certificates, leaving the registration to a separate board, representative of all the schools ; or whether (3) the registration of nurses should be undertaken by the managers of the Pension Fund, as Dr. Steele proposes—these are matters which the representative committee, to which the whole question has now been referred, will have to determine and report upon. In any case, all who desire to see nursing and nurses prosper, either in this country or elsewhere, will set their faces like flints against any proposals, by whomsoever made or patronised, which lead in the direction of a system of "Home Rule for Nurses." Disintegration of this character means disunion and disaster, and as we have said, the responsible authorities of all public institutions where nurses are trained or employed should combine, in the public interest and their own self-defence, to defeat every attempt on the part of a section of the staff under their control to force their hand in any direction whatever which they conceive to be hostile to the efficiency and prosperity of the undertakings under their management.

